# Effects of Vitamin D_3_ and Marine Omega-3 Fatty Acids Supplementation on Biomarkers of Systemic Inflammation: 4-Year Findings from the VITAL Randomized Trial

**DOI:** 10.3390/nu14245307

**Published:** 2022-12-14

**Authors:** Yanbin Dong, Haidong Zhu, Li Chen, Ying Huang, William Christen, Nancy R. Cook, Trisha Copeland, Samia Mora, Julie E. Buring, I-Min Lee, Karen H. Costenbader, JoAnn E. Manson

**Affiliations:** 1 Georgia Prevention Institute, Medical College of Georgia, Augusta University, Augusta, GA 30912, USA; 2 Department of Medicine, Brigham and Women’s Hospital, Harvard Medical School, Boston, MA 02115, USA; 3 Department of Epidemiology, Harvard T.H. Chan School of Public Health, Boston, MA 02115, USA

**Keywords:** vitamin D, marine *n*-3 fatty acids, inflammation

## Abstract

Background: The VITAL study was a nationwide, randomized, double-blind, placebo-controlled, 2 × 2 factorial trial of vitamin D_3_ (2000 IU/day) and marine *n*-3 FAs (1 g/day) supplements. We recently reported that vitamin D supplementation with or without omega 3 fatty acids reduced autoimmune disease by 22% in the VITAL study. Objective: To investigate the effects of vitamin D_3_ and/or *n*-3 FAs on changes in systemic inflammatory biomarkers including pro- and anti-inflammatory cytokines over a 4-year period in the VITAL sub-cohort with in-person evaluations at the Center for Clinical Investigations (CCI) in Boston. Design: Serum levels of four inflammatory biomarkers (high-sensitivity C-reactive protein [hs-CRP], interleukin-6, interleukin-10, and tumor necrosis factor-α) were measured in a total of 2713 samples from those 1054 VITAL/CCI participants (aged 64.9 ± 6.5 years, 49% female, 84% white, and 9% black) at baseline, year 2, and year 4 follow-up visits. Results: In multiple-adjusted models, vitamin D_3_ supplementation decreased serum hs-CRP levels by 19% at 2-year follow-up (nominal *p* = 0.007; *p*-value after multiple comparison adjustment = 0.028), but not at 4-year follow-up (nominal and adjusted *p*-values > 0.05). The effects of vitamin D_3_ on other inflammatory markers were not statistically significant either at year 2 or year 4 (all adjusted *p*-values > 0.05). Marine *n*-3 FAs were not significantly associated with changes of all the above inflammatory markers either at years 2 and 4, after multiple comparison adjustment (all *p*-values > 0.05). Conclusions: Vitamin D_3_ supplementation with or without *n*-3 FAs decreased hs-CRP by 19% at year 2, but not other inflammatory biomarkers at year 2 or year 4, while *n*-3 FAs with or without vitamin D_3_ did not significantly affect these biomarkers at either time point. Our findings support a potential role of vitamin D supplementation in modulating the chronic inflammatory process, systemic inflammation, and possibly autoimmune disease progression.

## 1. Introduction

Our recent reports from the large-scale primary prevention the VITamin D and OmegA-3 Trial (VITAL) trial have linked vitamin D supplementation to a reduced risk of autoimmune diseases [1]. Specifically, vitamin D supplementation with or without omega 3 fatty acids, reduced autoimmune disease by 22%, while omega 3 fatty acid supplementation with or without vitamin D reduced the autoimmune disease rate by 15% (not statistically significant) [1]. Systematic chronic inflammation plays an important role in the development of cancer [2,3] and autoimmune disorder [4], as well as cardiovascular disease (CVD) [5,6,7], type 2 diabetes [8,9,10], and numerous other chronic diseases [11,12,13]. In some previous randomized trials, vitamin D_3_ supplementation reduced high-sensitivity C-reactive protein (hs-CRP) in patients with diabetes [14], psychiatric disorders [15], and polycystic ovary syndrome [16], and reduced tumor necrosis factor-α (TNF-α) in patients with diabetes [17]. Marine omega-3 fatty acid (fish oil) supplementation has also been shown to reduce CRP in randomized trials among maintenance hemodialysis patients [18] and those with end-stage renal disease [19], and to reduce TNF-α and interleukin (IL)-6 among patients with diabetes [20] and chronic heart failure [21]. However, a systematic review of meta-analyses and randomized controlled trials (RCTs) found that vitamin D_3_ supplementation at a range of doses had no significant effect on biomarkers of systemic inflammation and hypothesized that low vitamin D status is a consequence of ill health, rather than its cause [22]. Similarly, another meta-analysis of RCTs with short intervention duration (2–6 months) found no difference in CRP, IL-6, or TNF-α between the omega-3 fatty acids (*n*-3 FAs) supplementation groups and the placebo groups [23]. Several meta-analyses point out the overall low quality of the evidence regarding vitamin D_3_ or *n*-3 FAs supplementation [22,24,25]. The conflicting findings may be due to small sample size, low doses, flawed study design, short intervention period, or publication bias. Therefore, a large and well-conducted RCT with adequate dosing and longer duration is needed to investigate the effects of vitamin D_3_ and/or *n*-3 FAs on markers of systemic inflammation.

VITAL is a recently completed randomized, double-blind, placebo-controlled trial of vitamin D_3_ (2000 IU/day) and marine *n*-3 FAs (1 g/day) for the primary prevention of cancer and CVDs among 25,871 women ≥ 55 and men ≥ 50 years of age with the mean treatment period of 5.3 years [26,27]. Costenbader et al. [28] previously reported that neither vitamin D_3_ nor *n*-3 FAs supplements significantly reduced systemic inflammation over 1 year among a subset of 1561 VITAL participants. Thus, in the current study, we aimed to investigate the effects of vitamin D_3_ and marine *n*-3 FAs supplementations on changes in biomarkers of systemic inflammation over 2-year and 4-year periods among the VITAL/CCI cohort (*N* = 1054) with in-person evaluations at the Center for Clinical Investigations (CCI) in Boston. Four inflammatory biomarkers, which included three pro-inflammatory markers (hs-CRP, IL-6, and TNF-α) and one anti-inflammatory cytokine (IL-10), were measured at baseline, 2-year, and 4-year follow-ups. Changes in the inflammatory biomarkers between baseline and 2 years or 4 years were evaluated for the interventional effects of vitamin D_3_ and/or marine *n*-3 FAs supplementations. For biomarkers affected by the supplements, the authors also explored possible subgroup-specific effects of vitamin D_3_ and marine *n*-3 FAs supplementations among categories of age, sex, race, body mass index (BMI), baseline nutrient status, and medications for high cholesterol, diabetes, or hypertension.

## 2. Subjects and Methods

### 2.1. Study Population

VITAL was a nationwide, randomized, double-blind, placebo-controlled trial of the benefits and risks of supplemental vitamin D_3_ (2000 IU/d) and marine *n*-3 FAs (1 g/d Omacor^®^ capsule with 840 mg of *n*-3 FAs, including eicosapentaenoic acid [EPA, 460 mg] + docosahexaenoic acid [DHA, 380 mg]) in the primary prevention of cancer and CVD among 25,871 U.S. men and women, aged ≥50 and ≥55, respectively [26,27]. Eligible participants had no history of cancer (except non-melanoma skin cancer), myocardial infarction, stroke, transient ischemic attack, or coronary revascularization. Participants were randomized to vitamin D_3_, *n*-3 FAs, both active agents, or both placebos in a 2 × 2 factorial design with 1:1:1:1 allocation ratio. Randomization was performed by the central coordinating center at BWH and participants were enrolled by study staff at BWH. Randomization was computer generated within sex, race, and 5-year age groups in blocks of eight. Everyone involved in the trial was blinded to interventions throughout the trial (participants, investigators, endpoint adjudicators, and care providers). The median intervention period was 5.3 years, with a range of 3.8 to 6.1 years. During the trial, yearly questionnaire response rates averaged 93%, and mortality follow-up rates exceeded 98%. The percentage of participants who reported taking at least two-thirds of their study capsules averaged 80% during the intervention period; and fewer than 5% and 10% of participants reported outside vitamin D_3_ use at 2 and 5 years, respectively [26]. VITAL and this ancillary study were registered at https://clinicaltrials.gov (accessed on 9 September 2016) (NCT01169259 and NCT04386577). All important harms were reported in the main paper [26,27]. The Partners’ Health Care System’s Institutional Review Board approved this ancillary study. All the participants provided written informed consent before enrollment in the VITAL trial.

### 2.2. Blood Collection and Laboratory Analysis

Boston-area participants (*N* = 1054) visited a local CCI clinic for detailed assessments and blood collections at baseline, year 2, and year 4 follow-up visits. Fasting blood samples were immediately processed and stored at −80 °C. hs-CRP, IL-6, IL-10, and TNF-α at baseline, year 2 and year 4 visits were measured in a total of 3000 serum samples including 287 quality control samples using Simple Plex assay, which was based on microfluidics and glass nanoreactor (GNR) technology (Simple Plex, Protein Simple Corp, San Jose, CA, USA). The immunoassays were developed using antibodies from R&D Systems (Minneapolis, MN, USA) reagents. The Ella platform automates the immunoassay by running samples in parallel through individual microfluidic channels, binding the protein of interest before washing off unbound analyte and adding a detection reagent. Each channel has three GNRs that are coated with a capture antibody so that results are produced in triplicate for each sample. The intra- and inter-assay variance of coefficients were 4.3% and 6.4% for hs-CRP, 3.9% and 7.1% for IL-6, 6.0% and 7.1% for IL-10, 4.9% and 4.5% for TNF-α. Among the 2713 study samples, 13 (0.5%) hs-CRP, 786 (29%) IL-6, 53 (2%) IL-10, and 3 (0.1%) TNF-α were outside the limits of quantification and were excluded from the statistical analysis.

### 2.3. Statistical Analysis

Analyses of effect were based on the intention-to-treat principle (all CCI participants who underwent randomization were included). The general characteristics of the participants are presented as mean ± standard deviation (SD) for continuous variables and *N* (%) for categorical variables. The normality of each continuous variable was tested based on a combination of test statistics of skewness and kurtosis, and a normalizing transformation was applied if necessary. Baseline differences of general characteristics between the placebo group and treatment group were tested using two-tailed t-test for continuous variables with normal distribution, Wilcoxon rank-sum test for non-normal distributed variables, and Pearson’s chi-squared for categorical variables.

The effects of vitamin D_3_ or/and marine *n*-3 FAs supplementation on changes in inflammatory markers were estimated by the interaction term of group assignment and time in two-level mixed-effects linear regression models with both fixed effects and participant-level random effects to handle repeated measurements at baseline, two years and four years. Autoregressive structure of order 1 of the within-group errors was assumed to account for successive observations within the groups. The inflammatory markers were log-transformed to reduce skewness of original data. Three models were constructed to control for potential confounding factor imbalance. Model 1 was adjusted for the randomization group for the other treatment; model 2 was adjusted for age, sex, race, and BMI, in addition to model 1; model 3 was adjusted for the covariates in model 2 and smoking, and taking medications for hypertension, diabetes, and high cholesterol. A *p* < 0.05 was considered nominally statistically significant. Due to multiple comparisons (4 biomarkers), a *p*-value of <0.01 (0.05/4 biomarkers) was considered statistically significant at each time point (2 and 4 years) for Bonferroni correction. Compliance-adjusted analyses were carried out by censoring follow-up data when the participant took less than 2/3 of study pills or began taking more than 800 IU per day of outside vitamin D or took outside use of fish-oil supplements. Similar statistical analyses were carried out to test the interactions of vitamin D_3_/*n*-3 FAs supplementation with age group, sex, race, smoking status, obesity, baseline nutrient status, and medications for high cholesterol, diabetes, or hypertension with regard to the inflammatory markers. Due to the number of comparisons, these results should be considered exploratory and interpreted with caution. All analyses were performed using Stata version 12.0 (StataCorp., College Station, TX, USA).

## 3. Results

### 3.1. General Characteristics of the Participants

A total of 1054 participants (aged 64.9 ± 6.5 years, 49% female, 84% white, and 9% black) were included in this study. A total of 2713 serum samples available for analysis (1051 samples were from baseline, 977 from year 2, and 685 from year 4). Table 1 presents the baseline characteristics according to vitamin D_3_ and marine *n*-3 FAs assignments. There was no significant difference in the baseline general characteristics between the vitamin D_3_ active group and vitamin D_3_ placebo group, or between the marine *n*-3 FAs active group and *n*-3 FAs placebo group (*p* > 0.05), except for a higher BMI in the active group than in the placebo group (*p* = 0.007).

### 3.2. Comparisons of Levels of Inflammatory Markers between the Placebo Group and Treatment Group

**Figure 1:**
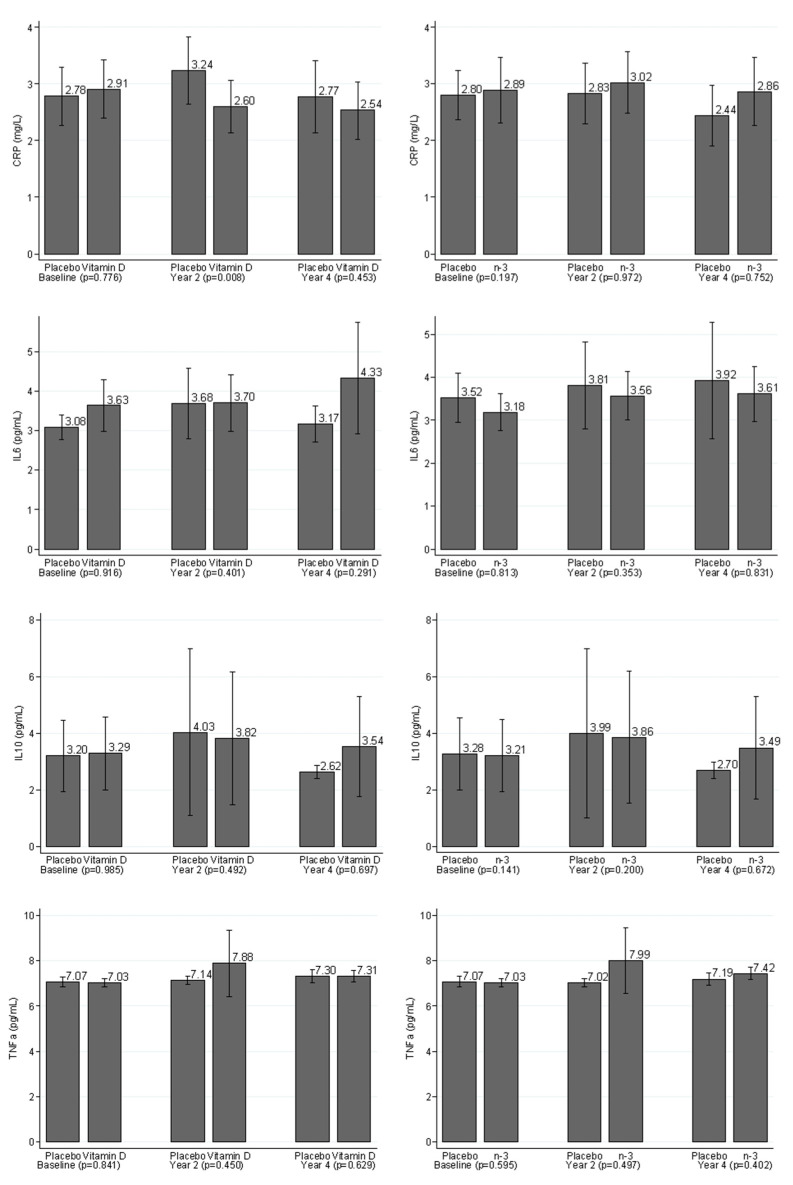
Bar charts of inflammatory markers grouped by vitamin D_3_ and *n*-3 FAs assignments at baseline, year 2, and year 4. Inflammatory markers were compared between the placebo group and treatment group on each visit as of baseline, year 2, and year 4; *p*-values were calculated using Wilcoxon rank-sum test because of the non-normal distribution of the inflammatory markers.

Levels of inflammatory markers were compared between the treatment group and the placebo group at each visit at baseline, year 2, and year 4 (Figure 1). Wilcoxon rank-sum tests indicated that hs-CRP levels were significantly lower in the vitamin D_3_ active group than vitamin D_3_ placebo group in year 2 (nominal *p* = 0.008; Bonferroni-adjusted *p* = 0.032), but other biomarkers did not differ significantly by vitamin D randomization at year 2. At year 4, no significant differences were apparent for vitamin D supplementation and any inflammatory biomarker (all nominal and adjusted *p*-values > 0.05). For marine *n*-3 FA supplementation, there were no significant differences in any inflammatory biomarker between the active and placebo groups at either year 2 or year 4 (all nominal and corrected *p*-values > 0.05).

### 3.3. Effects of Vitamin D_3_ Supplementation and Marine n-3 Fatty Acids on Changes in Markers of Inflammation in all Participants

Table 2 and Table 3 present the effects of vitamin D_3_ and marine *n*-3 FAs supplementation on inflammatory markers. Vitamin D_3_ supplementation decreased circulating hs-CRP levels at year 2 in all models, including the fully adjusted one (19% decrease, nominal *p* = 0.007; adjusted *p*-value = 0.028), but other inflammatory biomarkers were not significantly changed. At year 4, the reduction in hs-CRP was attenuated (nominal *p* = 0.753) and no other biomarkers were significantly changed.

Marine *n*-3 FAs supplementation did not significantly affect hs-CRP, IL-6, or IL-10 at year 2 or year 4 (all *p*-values > 0.05). Circulating TNF-α levels were slightly increased at year 2 (3% increase in multiple-adjusted model, nominal *p* = 0.049) and year 4 (4% increase in multiple-adjusted model, nominal *p* = 0.043), but the results were not significant after adjustment for multiple comparisons (adjusted *p* = values = 0.20 and 0.12, respectively).

### 3.4. Compliance-Adjusted Analysis

For vitamin D compliance-adjusted analyses, 5.3% and 9.4% of participants at year 2 and year 4 were excluded who took less than 2/3 study pills; 7.1% and 13.5% at year 2 and year 4 were also excluded who took more than 800 IU/day of outside vitamin D. For marine *n*-3 FA compliance-adjusted analyses, 4.6% and 8.8% participants at year 2 and year 4 were excluded who took less than 2/3 study pills; 1.0% and 1.7% at year 2 and year 4 were also excluded who took outside fish oil.

Table 4 and Table 5 present the results from compliance-adjusted analyses. When censoring the non-compliance participants, vitamin D_3_ supplementation decreased circulating hs-CRP levels at year 2 (nominal *p* = 0.027), but the results were not significant after adjustment for multiple comparisons (adjusted *p* = 0.11). Other inflammatory biomarkers were not significantly changed. At year 4, the reduction in hs-CRP was attenuated (nominal *p* = 0.898) and no other biomarkers were significantly changed. Marine *n*-3 FAs supplementation did not significantly affect hs-CRP, IL-6, or IL-10 at year 2 or year 4 (all *p*-values > 0.05). Circulating TNF-α levels were slightly increased at year 2 (nominal *p* = 0.037) and year 4 (nominal *p* = 0.035), but the results were not significant after adjustment for multiple comparisons (adjusted *p*-values = 0.15 and 0.14, respectively).

### 3.5. Exploratory Subgroup Analysis

Supplemental Figure S1 shows the results of the exploratory subgroup analyses of the associations between vitamin D_3_ supplementation and hs-CRP. At 2-year follow-up, vitamin D_3_ supplementation decreased hs-CRP among all subgroups, and the decreases were statistically significant among the younger group (age < median of 64.4 yrs, *p* = 0.018), males (*p* = 0.019), whites (*p* = 0.009), non-smokers (*p* = 0.018), baseline 25(OH)D ≥ 20 ng/mL (*p* = 0.005), higher baseline *n*-3 index (*p* = 0.012), and the ones that were not on medications for high cholesterol (*p* = 0.021), diabetes (*p* = 0.005), or hypertension (*p* = 0.015). However, *p*-values for interaction were not significant for any of the subgroups (all *p*-values > 0.05).

## 4. Discussion

In the VITAL sub-cohort with in-person evaluations at the Center for Clinical Investigations (CCI) in Boston, we found that vitamin D_3_ supplementation (2000 IU/day) decreased circulating levels of hs-CRP by 19% over a 2-year period, although the reduction was attenuated at 4 years. Other inflammatory biomarkers were not significantly altered by vitamin D_3_ and/or marine omega-3 fatty acids supplementation.

CRP is the most widely investigated biomarker of low-grade systemic inflammation [29] and a risk factor for CVD disease [11,30], cancer [31], autoimmune disorders [4], and several other chronic diseases. In the present study, vitamin D_3_ supplementation decreased circulating concentrations of hs-CRP over 2 years. Several small-scale and short-duration RCTs have tested the effect of vitamin D_3_ supplementation on lowering CRP, with both positive [32,33] and negative findings [34,35]. Fifty-one females with diabetes were randomly allocated to receive one oral pearl of 50,000 IU vitamin D_3_ (26 females) or a placebo (25 females) fortnightly for 16 weeks. Serum hs-CRP was significantly reduced, while IL-10 concentrations were increased in the intervention group [32]. In another study, 60 subjects, aged 18–40 years old with polycystic ovary syndrome, were randomly allocated to take either 50,000 IU vitamin D_3_ every 2 weeks plus 2000 mg/day *n*-3 FAs from fish oil (*n* = 30) or placebo (*n* = 30) for 12 weeks. Vitamin D_3_ and *n*-3 FAs co-supplementation significantly decreased hs-CRP [33]. In another study, 40 females with polycystic ovary syndrome were randomized to vitamin D_3_ (3200 IU) or placebo daily for 3 months. Vitamin D_3_ supplementation improved liver markers and insulin sensitivity, but had no effect on CRP [34]. In a post hoc analysis of 200 participants from a previous RCT, 1.25 mg vitamin D_3_ monthly supplementation (~1667 IU/day) for 24 months had no significant effect on changes in serum hs-CRP, IL-6, IL-8, IL-10, leptin, adiponectin, resistin, adipsin, and apelin [35]. Two systematic reviews and meta-analysis of RCTs showed that vitamin D_3_ supplementation significantly reduced hs-CRP, which is in agreement with our data [15,36]. VITAL is one of the largest RCTs of vitamin D_3_ supplementation conducted to date. In the present study, vitamin D_3_ decreased hs-CRP in the overall cohort and most subgroups at 2 years. Although the reduction was attenuated at 4 years, to what extent the 2-year “tempering” effect of vitamin D supplementation on hs-CRP influences the chronic inflammatory process, systemic inflammation, and autoimmune disease progression warrants further investigation. Our compliance-adjusted analyses showed that the attenuation was unlikely to be caused by compliance issues. Our sample was underpowered to look at the modifying effects of race/ethnicity, and this should be prioritized in future studies.

The underlying mechanism by which vitamin D_3_ may influence hs-CRP is not well understood. Vitamin D could decrease CRP via the MyD88/MAPK/NF-κB signaling pathway. An in vitro study showed that vitamin D exposure increased the expression of vitamin D receptors (VDR) in mast cells [37]. VDR formed complexes with Lyn in mast cells to inhibit the binding of Lyn to the β chain of FcεRI and MyD88, which decreased the levels of MAPK and its downstream marker NF-κB [37]. Another study showed that NF-κB inhibitor significantly inhibited CRP protein expression [38]. The main effects of vitamin D_3_ supplementation on other inflammatory markers (IL-6, IL-10, and TNF-α) were not significant. A previous VITAL ancillary study by Costenbader et al., reported that neither vitamin D_3_ nor *n*-3 FA supplementation over 1 year decreased markers of inflammation (hs-CRP, IL-6, and TNFR2), instead vitamin D_3_ supplementation increased IL-6 by 8.2% over 1 year [28]. There are several potential reasons that could explain the differences between their findings and ours. First, the two studies evaluated the effects of supplements over different follow-up durations. Costenbader’s study evaluated the effect at 1-year follow-up, and the current study evaluated the effect at 2 and 4-year follow-ups. Second, Costenbader’s study evaluated 1561 VITAL participants who had provided blood by overnight coolpack, with processing at ~24 h after collection. All the 1054 study participants in the present study were from the Boston CCI cohort, who had in-person visits, and their blood samples were processed immediately after blood draw using optimal procedures at baseline, 2, and 4 years.

Beneficial effects of *n*-3 FAs or fish oil on decreasing systemic inflammation have been found in previous studies with small sample sizes and short intervention periods, including TNF-α [39,40], IL-6 [41], CRP [39], and IL-8 [40]. We previously found no significant effect of *n*-3 FAs on hs-CRP, IL-6, or TNFR2 over 1-year period in the subset of the VITAL participants, but a signal for benefit was observed in African Americans [28]. A meta-analysis of 16 RCTs involving 1008 patients with gastrointestinal malignancy showed that *n*-3 FAs significantly reduced postoperative inflammatory markers, including IL-6, TNF-α, and CRP [41]. Other studies have shown no effects on inflammation [28,42]. A very recent systematic review and meta-analysis of 17 trials also concluded that walnuts, a rich source of α- linolenic acid and the plant-based *n*-3 FAs, did not statistically modified inflammatory markers, such as CRP, TNF-α, IL-6, IL-1β), in middle-aged and older adults [43]. In the present study, we found that marine *n*-3 FAs had no effect on hs-CRP and IL-6, but slightly increased TNF-α in year 4, a finding that did not persist after adjustment for multiple comparisons. In an animal study, fish oil increased mRNA expression levels of TNF-α, IL-1β, IL-6, IL-17, and IL-18 in colonic tissue compared to soybean oil [44]. The researchers postulated that the intake of fish oil might increase the taurine-conjugated bile acids, which promote the growth of proteobacteria, and the bacterium produce hydrogen sulfide to induce gut inflammation. In vitro studies showed that *n*-3 FAs might have dual effects on TNF-α. The anti-inflammatory effect was through the suppression of T-cell proliferation and pro-inflammatory cytokines secretion (IFN-γ) [45,46] and the inhibition of NF-κB/COX-2 induced production of pro-inflammatory [47]; whereas the pro-inflammatory effect was through resident peritoneal macrophages that increased lipopolysaccharide (LPS) and induced TNF-α secretion and decreased IL-10 secretion ex vivo [48,49].

Our study leveraged the large randomized, double-blind, placebo-controlled VITAL trial. Moreover, the effects of vitamin D_3_ and marine *n*-3 FAs supplementations on inflammation were evaluated for longer durations of 2 years and 4 years, which showed the dynamic responses to the supplementations longitudinally. We used biospecimens collected from the VITAL-CCI participants, who had in-person visits and blood samples were drawn on-site and optimally processed shortly after blood collection. Compared to the whole VITAL study, the CCI cohort had a lower percentage of African Americans participants. The present study also has several limitations. First, it was a post hoc analysis of an RCT, which was not originally designed to test the effect of vitamin D_3_ and marine *n*-3 FAs supplementations on biomarkers of inflammation. Second, the analyses involved multiple comparisons (4 biomarkers), thus increasing the likelihood of false positive findings. However, this concern was mitigated by multiple comparison adjustments. Third, results from subgroup analyses had limited power, were exploratory, and should be interpreted with caution. Fourth, it is possible that the outcomes of inflammation could have been altered if the participants would have received higher doses of vitamin D_3_. Two small short duration RCTs with high dose showed beneficial effects of vitamin D3 [50,51]. One study showed that high-dose vitamin D_3_ supplementation of 40,000 IU/week (~5700 IU/d) for 24 weeks was associated with improvement in clinical manifestation, cutaneous microcirculation and inflammatory markers (decreased IL-6 and increased IL-10) in patients with T2DM and peripheral neuropathy [50]. Another study showed that oral supplementation of vitamin D 3 (50,000 IU) once weekly (~7100 IU/d) for 12 weeks was associated with improvement in the serum level of vitamin D and significant decrease in the symptoms and sign of diabetic neuropathy [51]. However, RCTs of high-dose bolus dosing of vitamin D have been associated with several risks, including higher rates of fractures and falls, and long-term safety has not been documented [52,53].

## 5. Conclusions

In conclusion, vitamin D_3_ supplementation with or without *n*-3 FAs decreased hs-CRP by 19% at year 2, but not other inflammatory biomarkers at year 2 or year 4, while *n*-3 FAs with or without vitamin D3 did not significantly affect these biomarkers at either time point. Our findings support a potential role of vitamin D in modulating inflammation. Although the reduction was attenuated at 4 years, to what extent the 2-year “tempering” effect of vitamin D supplementation on hs-CRP intervenes the chronic inflammatory process, systemic inflammation, and autoimmune disease progression deserves further investigation. Whether the effect of vitamin D on hs-CRP mediates the observed associations between supplementation and lowered risks of autoimmune disorders also warrants future studies. More systemic inflammatory biomarkers should be included in the future studies as well. Although a large RCT with a higher dose of vitamin D_3_ with longer duration in the general population would be of interest, such a trial will require close monitoring for safety and the overall balance of benefits and risks.

## Figures and Tables

**Table 1:** Characteristics of the CCI participants at baseline according to vitamin D_3_ and marine *n*-3 FAs assignments.

Characteristic	Total (*N* = 1054)	Vitamin D_3_			*n*-3 FAs		
		Vitamin D_3_ (*N* = 520)	Placebo (*N* = 534)	*p*	*n*-3 FAs (*N* = 527)	Placebo (*N* = 527)	*p*
Age—yr	64.9 ± 6.5	64.7 ± 6.3	65.1 ± 6.6	0.600	64.8 ± 6.5	64.9 ± 6.4	0.777
Female sex—*N* (%)	515 (49)	256 (49)	259 (49)	0.813	260 (49)	255 (48)	0.758
Race—*N* (%)				0.622			0.390
White	871 (84)	429 (84)	442 (85)		429 (83)	442 (85)	
Black	88 (9)	42 (8)	46 (9)		43 (8)	45 (9)	
Others	73 (7)	40 (8)	33 (6)		42 (8)	31 (6)	
BMI—kg/m^2^	28.2 ± 5.3	28.1 ± 5.3	28.3 ± 5.4	0.296	28.7 ± 5.4	27.8 ± 5.3	0.007
Current smoking—*N* (%)	57 (5)	30 (6)	27 (5)	0.593	26 (5)	31 (6)	0.473
Medication use—*N* (%)							
Hypertension	432 (41)	204 (39)	228 (43)	0.253	220 (42)	212 (40)	0.616
Diabetes	89 (8)	49 (9)	40 (7)	0.259	45 (9)	44 (8)	0.912
Cholesterol	364 (35)	185 (36)	179 (34)	0.483	193 (37)	171 (32)	0.154
25(OH)D—ng/mL	28.1 ± 9.1	27.6 ± 8.8	28.7 ± 9.3	0.072	28.3 ± 9.5	28.0 ± 8.6	0.766
25(OH)D < 20 ng/mL—%	175 (17)	84 (16)	91 (17)	0.709	90 (17)	85 (16)	0.669
*n*−3 index—%	2.9 ± 1.0	2.9 ± 1.0	3.0 ± 1.0	0.472	3.0 ± 1.0	2.9 ± 1.0	0.441
SBP—mmHg	124.2 ± 14.3	123.7 ± 14.3	124.6 ± 14.6	0.325	124.2 ± 14.3	124.2 ± 14.6	0.837
DBP—mmHg	76.2 ± 9.2	75.8 ± 9.1	76.6 ± 9.3	0.092	76.6 ± 9.0	75.8 ± 9.4	0.163

Note: Statistics display as mean ± SD for continuous variables, and *N* (%) for categorical variables.

**Table 2:** Effects of vitamin D_3_ supplementation on log-transformed inflammatory markers.

	Ln hs-CRP		Ln IL-6		Ln IL-10		Ln TNF-α	
	β (SE)	*p*	β (SE)	*p*	β (SE)	*p*	β (SE)	*p*
Model 1	*N* = 2700		*N* = 1927		*N* = 2660		*N* = 2710	
Year 2	−0.16 (0.06)	0.010	−0.04 (0.05)	0.340	−0.00 (0.02)	0.887	0.00 (0.02)	0.878
Year 4	−0.02 (0.07)	0.762	0.06 (0.05)	0.272	−0.00 (0.03)	0.954	0.01 (0.02)	0.539
Model 2	*N* = 2647		*N* = 1882		*N* = 2607		*N* = 2657	
Year 2	−0.16 (0.06)	0.010	−0.05 (0.05)	0.269	−0.00 (0.02)	0.926	0.00 (0.02)	0.775
Year 4	−0.02 (0.07)	0.739	0.06 (0.05)	0.281	−0.00 (0.03)	0.999	0.01 (0.02)	0.497
Model 3	*N* = 2626		*N* = 1866		*N* = 2587		*N* = 2636	
Year 2	−0.17 (0.06)	0.007	−0.05 (0.05)	0.267	−0.00 (0.02)	0.962	0.00 (0.02)	0.837
Year 4	−0.02 (0.07)	0.753	0.06 (0.05)	0.292	0.00 (0.03)	0.907	0.01 (0.02)	0.495

Note: Effects of vitamin D_3_ supplementation on cytokines was estimated by the interaction term of group assignment and measure time in two-level mixed-effects linear regression models. Autoregressive structure of order 1 of the within-group errors was assumed to account for successive observations with the groups. The inflammatory markers were log-transformed. Model 1 was adjusted for the randomization group for the other treatment; model 2 was adjusted for age, sex, race, and BMI in addition to model 1; model 3 was adjusted for the covariates in model 2 and smoking status, and taking medications for hypertension, diabetes, or lowing cholesterol. A *p*-value of <0.01 was considered statistically significant for Bonferroni correction.

**Table 3:** Effects of marine *n*-3 fatty acids on log-transformed inflammatory markers.

	hs-CRP		IL-6		IL-10		TNF-α	
	β (SE)	*p*	β (SE)	*p*	β (SE)	*p*	β (SE)	*p*
Model 1	*N* = 2700		*N* = 1927		*N* = 2660		*N* = 2710	
Year 2	−0.06 (0.06)	0.330	0.04 (0.05)	0.357	0.03 (0.02)	0.160	0.03 (0.02)	0.076
Year 4	−0.04 (0.07)	0.631	0.04 (0.05)	0.459	0.01 (0.03)	0.661	0.04 (0.02)	0.035
Model 2	*N* = 2647		*N* = 1882		*N* = 2607		*N* = 2657	
Year 2	−0.06 (0.06)	0.336	0.06 (0.05)	0.245	0.03 (0.02)	0.145	0.03 (0.02)	0.054
Year 4	−0.03 (0.07)	0.637	0.05 (0.05)	0.367	0.01 (0.03)	0.653	0.04 (0.02)	0.037
Model 3	*N* = 2626		*N* = 1866		*N* = 2587		*N* = 2636	
Year 2	−0.05 (0.06)	0.445	0.05 (0.05)	0.258	0.03 (0.02)	0.140	0.03 (0.02)	0.049
Year 4	−0.04 (0.07)	0.632	0.05 (0.05)	0.352	0.01 (0.03)	0.648	0.04 (0.02)	0.043

Note: Effects of marine *n*-3 FAs supplementation on cytokines was estimated by the interaction term of group assignment and measure time in two-level mixed-effects linear regression models. Autoregressive structure of order 1 of the within-group errors was assumed to account for successive observations with the groups. The inflammatory markers were log-transformed. Model 1 was adjusted for the randomization group for the other treatment; model 2 was adjusted for age, sex, race, and BMI in addition to model 1; model 3 was adjusted for the covariates in model 2 and smoking status, and taking medications for hypertension, diabetes, and high cholesterol. A *p*-value of <0.01 was considered statistically significant for Bonferroni correction.

**Table 4:** Compliance-adjusted analysis of vitamin D_3_ supplementation on log-transformed inflammatory markers.

	hs-CRP		IL-6		IL-10		TNF-α	
	β (SE)	*p*	β (SE)	*p*	β (SE)	*p*	β (SE)	*p*
Model 1	*N* = 2530		*N* = 1795		*N* = 2492		*N* = 2540	
Year 2	−0.13 (0.06)	0.033	−0.07 (0.05)	0.184	0.00 (0.02)	0.835	0.00 (0.02)	0.829
Year 4	0.01 (0.08)	0.945	0.06 (0.06)	0.268	−0.01 (0.03)	0.718	0.01 (0.02)	0.813
Model 2	*N* = 2482		*N* = 1755		*N* = 2444		*N* = 2492	
Year 2	−0.13 (0.06)	0.043	−0.07 (0.05)	0.160	0.00 (0.02)	0.843	0.01 (0.02)	0.718
Year 4	0.01 (0.08)	0.912	0.07 (0.06)	0.253	−0.01 (0.03)	0.773	0.01 (0.02)	0.725
Model 3	*N* = 2461		*N* = 1739		*N* = 2424		*N* = 2471	
Year 2	−0.14 (0.06)	0.027	−0.07 (0.05)	0.149	0.01 (0.02)	0.824	0.00 (0.02)	0.809
Year 4	0.01 (0.08)	0.898	0.07 (0.06)	0.258	−0.00 (0.03)	0.878	0.01 (0.02)	0.713

Note: Effects of vitamin D_3_ supplementation on cytokines was estimated by the interaction term of group assignment and measure time in two-level mixed-effects linear regression models. Autoregressive structure of order 1 of the within-group errors was assumed to account for successive observations with the groups. The inflammatory markers were log-transformed. Model 1 was adjusted for the randomization group for the other treatment; model 2 was adjusted for age, sex, race, and BMI in addition to model 1; model 3 was adjusted for the covariates in model 2 and smoking status, and taking medications for hypertension, diabetes, or lowing cholesterol. A *p*-value of <0.01 was considered statistically significant for Bonferroni correction.

**Table 5:** Compliance-adjusted analysis of marine *n*-3 fatty acids on log-transformed inflammatory markers.

	hs-CRP		IL-6		IL-10		TNF-α	
	β (SE)	*p*	β (SE)	*p*	β (SE)	*p*	β (SE)	*p*
Model 1	*N* = 2533		*N* = 1873		*N* = 2593		*N* = 2643	
Year 2	−0.06 (0.06)	0.342	0.05 (0.05)	0.345	0.03 (0.02)	0.126	0.03 (0.02)	0.054
Year 4	−0.04 (0.07)	0.577	0.06 (0.06)	0.307	0.01 (0.03)	0.628	0.05 (0.02)	0.027
Model 2	*N* = 2585		*N* = 1833		*N* = 2545		*N* = 2595	
Year 2	−0.06 (0.06)	0.360	0.06 (0.05)	0.228	0.04 (0.02)	0.116	0.04 (0.02)	0.041
Year 4	−0.04 (0.07)	0.604	0.06 (0.06)	0.245	0.01 (0.03)	0.628	0.05 (0.02)	0.029
Model 3	*N* = 2564		*N* = 1817		*N* = 2525		*N* = 2574	
Year 2	−0.04 (0.06)	0.475	0.06 (0.05)	0.239	0.04 (0.02)	0.110	0.04 (0.02)	0.037
Year 4	−0.04 (0.08)	0.595	0.07 (0.06)	0.231	0.01 (0.03)	0.621	0.04 (0.02)	0.035

Note: Effects of marine *n*-3 FAs supplementation on cytokines was estimated by the interaction term of group assignment and measure time in two-level mixed-effects linear regression models. Autoregressive structure of order 1 of the within-group errors was assumed to account for successive observations with the groups. The inflammatory markers were log-transformed. Model 1 was adjusted for the randomization group for the other treatment; model 2 was adjusted for age, sex, race, and BMI in addition to model 1; model 3 was adjusted for the covariates in model 2 and smoking status, and taking medications for hypertension, diabetes, and high cholesterol. A *p*-value of <0.01 was considered statistically significant for Bonferroni correction.

## Data Availability

Data described in the manuscript, codebook, and analytic code will be made available upon request pending application to the corresponding author.

## Supplementary Materials

The following supporting information can be downloaded at: https://www.mdpi.com/article/10.3390/nu14245307/s1, Figure S1: Subgroup analysis for the effects of vitamin D3 supplementation on hs-CRP at year 2.

## Clinical Trial Registry

This trial was registered at https://clinicaltrials.gov (accessed on 9 September 2016) as NCT04386577.

## Abbreviations

RCT: randomized controlled trial; *n*-3 FAs: omega-3 fatty acids; MI: myocardial infarction; CHD: coronary heart disease; CVD: cardiovascular disease; hs-CRP: high sensitivity C-reactive protein; IL: interleukin; TNF-α: tumor necrosis factor-α; VITAL: VITamin D and OmegA-3 TriaL; CCI: Center for Clinical Investigations; 25(OH)D: 25-hydroxyvitamin D; TNFR2: tumor necrosis factor receptor 2; EPA: eicosapentaenoic acid; DHA: docosahexaenoic acid; GNR: glass nanoreactor; SD: standard deviation; BMI: body mass index; COVID-19: coronavirus disease 2019; RR: relative risk; VDR: vitamin D receptor; LPS: lipopolysaccharide.
