# Supplementary material for: Effects of Vitamin D3 and Marine Omega-3 Fatty Acids Supplementation on Biomarkers of Systemic Inflammation: 4-Year Findings from the VITAL Randomized Trial
Source: Nutrients. 2022 Dec 14;14(24):5307. doi: 10.3390/nu14245307 (PMC9782648; doi:10.3390/nu14245307)
Supplement: Supplementary file 1 [file nutrients-14-05307-s001.zip › nutrients-2050811 -supplementary.pdf]

Online Supporting Material

Effect of vitamin D supplementation on hs-CRP (year 2)

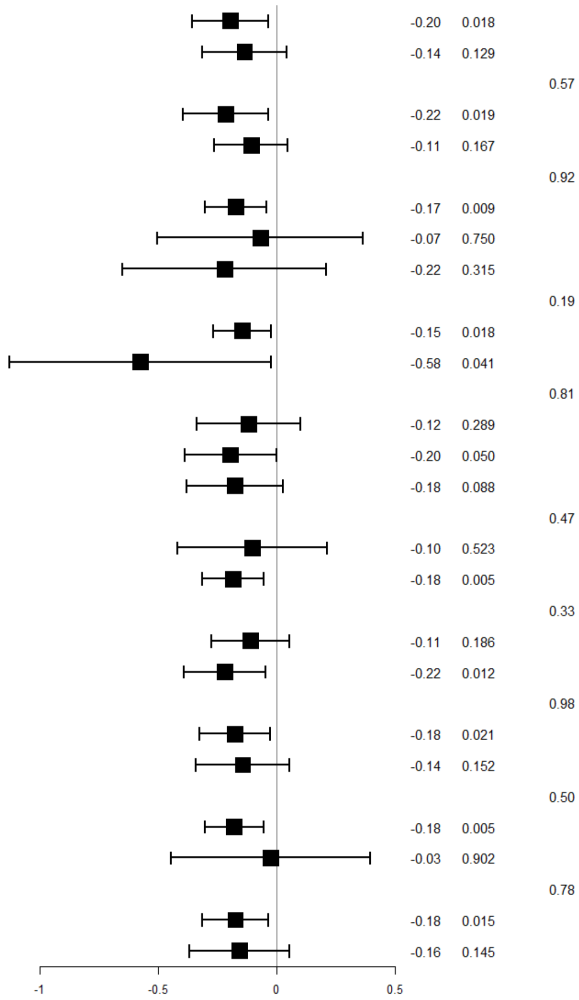

	N	Beta	p-value	P for interaction
Age				0.64
<Median of 64.4 yr	516	-0.20	0.018	
>=Median of 64.4 yr	509	-0.14	0.129	
Sex				0.57
Male	523	-0.22	0.019	
Female	502	-0.11	0.167	
Race				0.92
Non-Hispanic white	867	-0.17	0.009	
Black	85	-0.07	0.750	
Other	73	-0.22	0.315	
Current smoker				0.19
No	971	-0.15	0.018	
Yes	54	-0.58	0.041	
BMI				0.81
<25	300	-0.12	0.289	
25-30	428	-0.20	0.050	
30+	297	-0.18	0.088	
Baseline 25(OH)D				0.47
<20 ng/ml	170	-0.10	0.523	
>=20 ng/ml	854	-0.18	0.005	
Baseline omega-3 index				0.33
<Median of 2.7%	458	-0.11	0.186	
>=Median of 2.7%	563	-0.22	0.012	
Cholesterol medication				0.98
No	670	-0.18	0.021	
Yes	355	-0.14	0.152	
Diabetes medication				0.50
No	937	-0.18	0.005	
Yes	88	-0.03	0.902	
Hypertension medication				0.78
No	601	-0.18	0.015	
Yes	424	-0.16	0.145	

Online Supporting Material

Supplemental Figure S1. Subgroup analysis for the effects of vitamin D_3 supplementation on hs-CRP at year 2. The effects of vitamin D_3 supplementation on hs-CRP were estimated by the interaction term of group assignment and measure time in two-level mixed-effects linear regression models. Autoregressive structure of order 1 of the within-group errors was assumed to account for successive observations with the groups. The hs-CRP was log-transformed. Models were adjusted for n-3 fatty acid randomization group (intention-to-treat analysis), age, sex, race, BMI, smoking, and taking medications for hypertension, diabetes, and lowing high cholesterol.
